# 2-[(*E*)-(Dimethyl­amino)methyl­ene­amino]benzonitrile

**DOI:** 10.1107/S1600536809015979

**Published:** 2009-05-14

**Authors:** Xia Wei, Daxin Shi, Yanqiu Fan, Ling Zhang, Jiarong Li

**Affiliations:** a School of Chemical Engineering and Environment, Beijing Institute of Technology, Beijing 100081, People’s Republic of China

## Abstract

In the title compound, C_10_H_11_N_3_, the amidine unit, including the two methyl substituents, is virtually planar [maximum deviation = 0.016 (5) Å]. The plane of the benzene ring forms a dihedral angle of 46.5 (3)° with the amidine group.

## Related literature

For application of formamidines in chemical synthesis, see: Deshpande & Seshadri (1973 ▶); Toste *et al.* (1994 ▶).
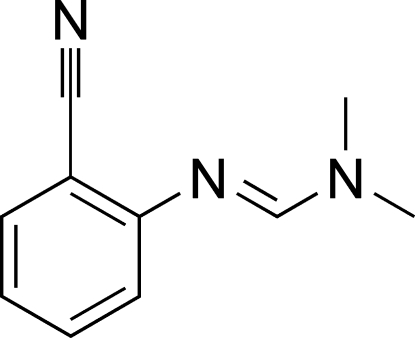


## Experimental

### 

#### Crystal data

C_10_H_11_N_3_
*M* *_r_* = 173.22
Monoclinic, 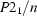

*a* = 7.7468 (15) Å
*b* = 11.212 (2) Å
*c* = 11.042 (2) Å
β = 109.67 (3)°
*V* = 903.1 (3) Å^3^
*Z* = 4
Mo *K*α radiation
μ = 0.08 mm^−1^
*T* = 113 K
0.20 × 0.18 × 0.14 mm

#### Data collection

Rigaku Saturn diffractometer
Absorption correction: multi-scan (*CrystalClear*; Rigaku/MSC, 2005 ▶) *T*_min_ = 0.984, *T*_max_ = 0.989
5746 measured reflections
1575 independent reflections
1342 reflections with *I* > 2σ(*I*)
*R*_int_ = 0.027

#### Refinement

*R*[*F* ^2^ > 2σ(*F* ^2^)] = 0.035
*wR*(*F* ^2^) = 0.100
*S* = 1.10
1575 reflections
120 parameters
H-atom parameters constrained
Δρ_max_ = 0.19 e Å^−3^
Δρ_min_ = −0.17 e Å^−3^

### 

Data collection: *CrystalClear* (Rigaku/MSC, 2005 ▶); cell refinement: *CrystalClear*; data reduction: *CrystalClear*; program(s) used to solve structure: *SHELXS97* (Sheldrick, 2008 ▶); program(s) used to refine structure: *SHELXL97* (Sheldrick, 2008 ▶); molecular graphics: *SHELXTL* (Sheldrick, 2008 ▶); software used to prepare material for publication: *SHELXTL*.

## Supplementary Material

Crystal structure: contains datablocks global, I. DOI: 10.1107/S1600536809015979/gk2203sup1.cif

Structure factors: contains datablocks I. DOI: 10.1107/S1600536809015979/gk2203Isup2.hkl

Additional supplementary materials:  crystallographic information; 3D view; checkCIF report

## Figures and Tables

**Fig. 1.**
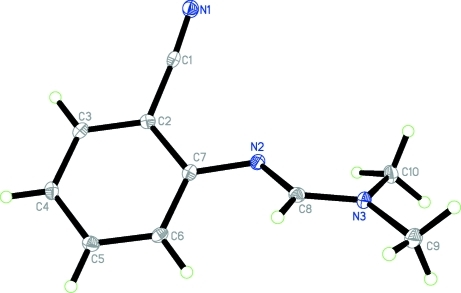
Molecular structure of the title compound with displacement ellipsoids drawn at the 30% probability level.

## supplementary crystallographic information

### Comment

Derivatives of formamidine are valuable synthetic intermediates featuring common structural motif found in a variety of compounds with interesting medicinal and biological properties. The formamidine group is a useful primary amine protecting group for its ease of introduction and efficient removal (Toste *et al.*, 1994). The *N*'-(2-cyanophenyl)-*N*,*N*-dimethylformamidine compounds are key intermediates of convenient synthesis of *O*-aminobenzonitrile, 4-aminoquinazolines and 4-aminoquinazoline-3-oxides (Deshpande *et al.*, 1973).

### Experimental

Phosphorus oxychloride (13 mmole) was added dropwise to 8 ml dimethylformamide at 273 K. After stirring for 2–3 min, finely powdered isatin-3-oxime (10 mmol) was added and  kept at room temperature for some time. Temperature was then gradually raised to 343 K and the reaction mixture was kept at this temperature for 2hr, then cooled, poured onto crushed ice and filtered. The clear filtrate was basified by sodium carbonate to pH=9 and the solution extracted with toluene, which was then evaporated to obtain crude (*E*)-*N*'-(2-cyanophenyl)-*N*,*N*-dimethylformamidine. The compound was recrystallizated from ethyl acetate and petroleum ether to give colorless crystals.

m.p. 338–339 K; IR(KBr): 2910 (C—H), 2214.87 (–CN), 1587, 1556 (C—C), 1367 (–CH3) cm^-1^; 1*H*-NMR (CDCl3, p.p.m): 3.07–3.09 (6*H*, m), 6.93–7.02(2*H*, m), 7.38–7.44 (1*H*, t), 7.51–7.54 (1*H*, d), 7.58 (1*H*, s); ESI: 174.1[*M*+H]^+^. Elementary analysis: found N 24.31, C 69.31, H 6.30; calc. 24.26, 69.34, 6.40).

20 mg of the obtained product was dissolved in ethyl acetate (5 ml). Then petroleum ether (2 ml) was added dropwise to the solution. The solution was kept at room temperature for 4 days to give colorless single crystals.

### Refinement

All H atoms were included in calculated positions and refined in the riding model approximation with C—H distances 0.93 (aromatic) or 0.96 Å (methyl), and with *U*_iso_=1.2*U*_eq_ or 1.5*U*_eq_(methyl).

### Crystal data

**Table:**

C_10_H_11_N_3_	*F*(000) = 368
*M**_r_* = 173.22	*D*_x_ = 1.274 Mg m^−^^3^
Monoclinic, *P*2_1_/*n*	Mo *K*α radiation, λ = 0.71073 Å
Hall symbol: -p 2yn	Cell parameters from 2901 reflections
*a* = 7.7468 (15) Å	θ = 1.8–27.9°
*b* = 11.212 (2) Å	µ = 0.08 mm^−^^1^
*c* = 11.042 (2) Å	*T* = 113 K
β = 109.67 (3)°	Cube, colorless
*V* = 903.1 (3) Å^3^	0.20 × 0.18 × 0.14 mm
*Z* = 4	

### Data collection

**Table:**

Rigaku Saturn diffractometer	1575 independent reflections
Radiation source: rotating anode	1342 reflections with *I* > 2σ(*I*)
confocal	*R*_int_ = 0.027
ω scans	θ_max_ = 25.0°, θ_min_ = 2.7°
Absorption correction: multi-scan (*CrystalClear*; Rigaku/MSC, 2005)	*h* = −9→8
*T*_min_ = 0.984, *T*_max_ = 0.989	*k* = −13→13
5746 measured reflections	*l* = −13→13

### Refinement

**Table:**

Refinement on *F*^2^	Primary atom site location: structure-invariant direct methods
Least-squares matrix: full	Secondary atom site location: difference Fourier map
*R*[*F*^2^ > 2σ(*F*^2^)] = 0.035	Hydrogen site location: inferred from neighbouring sites
*wR*(*F*^2^) = 0.100	H-atom parameters constrained
*S* = 1.10	*w* = 1/[σ^2^(*F*_o_^2^) + (0.0612*P*)^2^ + 0.137*P*] where *P* = (*F*_o_^2^ + 2*F*_c_^2^)/3
1575 reflections	(Δ/σ)_max_ = 0.001
120 parameters	Δρ_max_ = 0.19 e Å^−^^3^
0 restraints	Δρ_min_ = −0.17 e Å^−^^3^

### Special details

**Table:**

Geometry. All e.s.d.'s (except the e.s.d. in the dihedral angle between two l.s. planes) are estimated using the full covariance matrix. The cell e.s.d.'s are taken into account individually in the estimation of e.s.d.'s in distances, angles and torsion angles; correlations between e.s.d.'s in cell parameters are only used when they are defined by crystal symmetry. An approximate (isotropic) treatment of cell e.s.d.'s is used for estimating e.s.d.'s involving l.s. planes.
Refinement. Refinement of *F*^2^ against ALL reflections. The weighted *R*-factor *wR* and goodness of fit *S* are based on *F*^2^, conventional *R*-factors *R* are based on *F*, with *F* set to zero for negative *F*^2^. The threshold expression of *F*^2^ > σ(*F*^2^) is used only for calculating *R*-factors(gt) *etc*. and is not relevant to the choice of reflections for refinement. *R*-factors based on *F*^2^ are statistically about twice as large as those based on *F*, and *R*- factors based on ALL data will be even larger.

### Fractional atomic coordinates and isotropic or equivalent isotropic displacement parameters (Å^2^)

**Table:**

	*x*	*y*	*z*	*U*_iso_*/*U*_eq_	
N1	−0.14481 (15)	0.99786 (9)	0.10732 (10)	0.0224 (3)	
N2	0.07129 (13)	0.79099 (9)	0.36257 (9)	0.0164 (3)	
N3	0.06421 (14)	0.76114 (9)	0.56842 (9)	0.0188 (3)	
C1	−0.06597 (16)	0.90883 (10)	0.12357 (11)	0.0161 (3)	
C2	0.02451 (15)	0.79498 (10)	0.13459 (11)	0.0149 (3)	
C3	0.04571 (16)	0.74551 (11)	0.02435 (11)	0.0175 (3)	
H3	0.0069	0.7875	−0.0527	0.021*	
C4	0.12454 (16)	0.63393 (11)	0.02985 (12)	0.0189 (3)	
H4	0.1380	0.6005	−0.0435	0.023*	
C5	0.18342 (16)	0.57226 (11)	0.14586 (11)	0.0177 (3)	
H5	0.2354	0.4970	0.1496	0.021*	
C6	0.16547 (15)	0.62165 (10)	0.25572 (11)	0.0163 (3)	
H6	0.2079	0.5795	0.3326	0.020*	
C7	0.08444 (16)	0.73434 (11)	0.25378 (11)	0.0145 (3)	
C8	0.04348 (16)	0.72373 (11)	0.45021 (11)	0.0170 (3)	
H8	0.0071	0.6452	0.4290	0.020*	
C9	0.02345 (19)	0.68383 (12)	0.66108 (13)	0.0272 (3)	
H9A	−0.0296	0.6108	0.6197	0.041*	
H9B	−0.0615	0.7231	0.6941	0.041*	
H9C	0.1345	0.6666	0.7305	0.041*	
C10	0.13692 (18)	0.87915 (11)	0.61196 (12)	0.0231 (3)	
H10A	0.2098	0.9060	0.5621	0.035*	
H10B	0.2115	0.8756	0.7011	0.035*	
H10C	0.0374	0.9337	0.6014	0.035*	

### Atomic displacement parameters (Å^2^)

**Table:**

	*U*^11^	*U*^22^	*U*^33^	*U*^12^	*U*^13^	*U*^23^
N1	0.0285 (6)	0.0188 (6)	0.0200 (6)	0.0012 (5)	0.0085 (5)	0.0002 (4)
N2	0.0166 (5)	0.0180 (5)	0.0143 (5)	0.0000 (4)	0.0048 (4)	0.0003 (4)
N3	0.0216 (6)	0.0221 (6)	0.0146 (6)	0.0030 (4)	0.0086 (4)	0.0022 (4)
C1	0.0171 (6)	0.0187 (6)	0.0116 (6)	−0.0043 (5)	0.0038 (5)	−0.0009 (5)
C2	0.0126 (6)	0.0145 (6)	0.0169 (6)	−0.0025 (5)	0.0038 (5)	−0.0002 (4)
C3	0.0174 (6)	0.0197 (6)	0.0138 (6)	−0.0019 (5)	0.0033 (5)	0.0018 (5)
C4	0.0195 (6)	0.0207 (6)	0.0163 (6)	−0.0019 (5)	0.0057 (5)	−0.0045 (5)
C5	0.0156 (6)	0.0154 (6)	0.0210 (6)	−0.0001 (5)	0.0044 (5)	−0.0014 (5)
C6	0.0146 (6)	0.0171 (6)	0.0154 (6)	−0.0023 (5)	0.0025 (5)	0.0027 (5)
C7	0.0110 (6)	0.0171 (6)	0.0145 (6)	−0.0049 (4)	0.0029 (5)	−0.0011 (4)
C8	0.0145 (6)	0.0180 (6)	0.0184 (7)	0.0018 (5)	0.0053 (5)	0.0008 (5)
C9	0.0312 (7)	0.0328 (8)	0.0228 (7)	0.0074 (6)	0.0159 (6)	0.0092 (6)
C10	0.0243 (7)	0.0269 (7)	0.0182 (7)	0.0031 (6)	0.0071 (5)	−0.0046 (5)

### Geometric parameters (Å, °)

**Table:**

N1—C1	1.1522 (15)	C4—H4	0.9300
N2—C8	1.3007 (15)	C5—C6	1.3825 (16)
N2—C7	1.3925 (15)	C5—H5	0.9300
N3—C8	1.3283 (15)	C6—C7	1.4077 (17)
N3—C9	1.4544 (15)	C6—H6	0.9300
N3—C10	1.4549 (16)	C8—H8	0.9300
C1—C2	1.4415 (16)	C9—H9A	0.9600
C2—C3	1.3967 (16)	C9—H9B	0.9600
C2—C7	1.4137 (17)	C9—H9C	0.9600
C3—C4	1.3844 (17)	C10—H10A	0.9600
C3—H3	0.9300	C10—H10B	0.9600
C4—C5	1.3906 (17)	C10—H10C	0.9600
			
C8—N2—C7	117.12 (10)	C7—C6—H6	119.3
C8—N3—C9	121.34 (11)	N2—C7—C6	123.91 (11)
C8—N3—C10	121.14 (10)	N2—C7—C2	119.17 (11)
C9—N3—C10	117.46 (10)	C6—C7—C2	116.79 (10)
N1—C1—C2	175.84 (12)	N2—C8—N3	123.54 (11)
C3—C2—C7	121.49 (11)	N2—C8—H8	118.2
C3—C2—C1	118.33 (10)	N3—C8—H8	118.2
C7—C2—C1	120.15 (10)	N3—C9—H9A	109.5
C4—C3—C2	120.01 (11)	N3—C9—H9B	109.5
C4—C3—H3	120.0	H9A—C9—H9B	109.5
C2—C3—H3	120.0	N3—C9—H9C	109.5
C3—C4—C5	119.52 (11)	H9A—C9—H9C	109.5
C3—C4—H4	120.2	H9B—C9—H9C	109.5
C5—C4—H4	120.2	N3—C10—H10A	109.5
C6—C5—C4	120.70 (11)	N3—C10—H10B	109.5
C6—C5—H5	119.7	H10A—C10—H10B	109.5
C4—C5—H5	119.7	N3—C10—H10C	109.5
C5—C6—C7	121.48 (11)	H10A—C10—H10C	109.5
C5—C6—H6	119.3	H10B—C10—H10C	109.5
			
C7—C2—C3—C4	0.94 (17)	C5—C6—C7—C2	−0.75 (16)
C1—C2—C3—C4	−177.12 (10)	C3—C2—C7—N2	175.72 (9)
C2—C3—C4—C5	−0.49 (17)	C1—C2—C7—N2	−6.25 (16)
C3—C4—C5—C6	−0.57 (17)	C3—C2—C7—C6	−0.32 (16)
C4—C5—C6—C7	1.21 (17)	C1—C2—C7—C6	177.71 (10)
C8—N2—C7—C6	−35.50 (15)	C7—N2—C8—N3	166.09 (11)
C8—N2—C7—C2	148.77 (11)	C9—N3—C8—N2	177.22 (10)
C5—C6—C7—N2	−176.58 (10)	C10—N3—C8—N2	−5.75 (18)
